# Endurance Performance during Severe-Intensity Intermittent Cycling: Effect of Exercise Duration and Recovery Type

**DOI:** 10.3389/fphys.2016.00602

**Published:** 2016-12-02

**Authors:** Luis F. Barbosa, Benedito S. Denadai, Camila C. Greco

**Affiliations:** Human Performance Laboratory, Biosciences Institute, São Paulo State UniversityRio Claro, Brazil

**Keywords:** aerobic, oxygen uptake, passive, active, exercise tolerance

## Abstract

Slow component of oxygen uptake (VO_2_SC) kinetics and maximal oxygen uptake (VO_2_max) attainment seem to influence endurance performance during constant-work rate exercise (CWR) performed within the severe intensity domain. In this study, it was hypothesized that delaying the attainment of VO_2_max by reducing the rates at which VO_2_ increases with time (VO_2_SC kinetics) would improve the endurance performance during severe-intensity intermittent exercise performed with different work:recovery duration and recovery type in active individuals. After the estimation of the parameters of the VO_2_SC kinetics during CWR exercise, 18 males were divided into two groups (Passive and Active recovery) and performed at different days, two intermittent exercises to exhaustion (at 95% IVO_2_max, with work: recovery ratio of 2:1) with the duration of the repetitions calculated from the onset of the exercise to the beginning of the VO_2_SC (Short) or to the half duration of the VO_2_SC (Long). The active recovery was performed at 50% IVO_2_max. The endurance performance during intermittent exercises for the Passive (Short = 1523 ± 411; Long = 984 ± 260 s) and Active (Short = 902 ± 239; Long = 886 ± 254 s) groups was improved compared with CWR condition (Passive = 540 ± 116; Active = 489 ± 84 s). For Passive group, the endurance performance was significantly higher for Short than Long condition. However, no significant difference between Short and Long conditions was found for Active group. Additionally, the endurance performance during Short condition was higher for Passive than Active group. The VO_2_SC kinetics was significantly increased for CWR (Passive = 0.16 ± 0.04; Active = 0.16 ± 0.04 L.min^−2^) compared with Short (Passive = 0.01 ± 0.01; Active = 0.03 ± 0.04 L.min^−2^) and Long (Passive = 0.02 ± 0.01; Active = 0.01 ± 0.01 L.min^−2^) intermittent exercise conditions. No significant difference was found among the intermittent exercises. It can be concluded that the endurance performance is negatively influenced by active recovery only during shorter high-intensity intermittent exercise. Moreover, the improvement in endurance performance seems not be explained by differences in the VO_2_SC kinetics, since its values were similar among all intermittent exercise conditions.

## Introduction

The parameters of the power-time relationship, termed critical power (CP) and the curvature constant (W'), have been used to analyze the physiological responses and endurance performance during high-intensity exercise (Poole et al., 1988). CP has been considered the lower boundary of the severe-intensity domain and the W' determines the amount of external work that can be performed above CP, irrespective of the rate of its expenditure (Jones et al., 2010). By definition, all severe-intensity work rates (i.e., >CP) performed until voluntary exhaustion drive pulmonary oxygen uptake (VO_2_) to a maximal value (i.e., maximal oxygen uptake—VO_2_max) (Jones et al., 2010). However, during exhaustive exercise performed above the upper bound of the severe intensity domain, exercise duration would be too short to permit attainment of VO_2_max Caputo and Denadai (2008). Several studies have demonstrated that endurance exercise performance within severe-intensity domain was coincident with the depletion of the W', accumulation of metabolites associated with fatigue (i.e., PCr, Pi, and H^+^), and attainment of VO_2_max due to VO_2_ slow component (VO_2_SC) development (Fukuba et al., 2003; Chidnok et al., 2013). Indeed, VO_2_SC has been associated with loss in muscular efficiency (Jones et al., 2011) and has been negatively related with endurance performance (Zoladz et al., 1995; Murgatroyd et al., 2011; Barbosa et al., 2014a).

VO_2_ kinetics and muscle [PCr] responses to high-intensity exercise have been reported to present both fundamental and slow component phases (Rossiter et al., 2002) being intrinsically linked. Indeed, Rossiter et al. (2002) have reported similar values of the time constant (τ) of the fundamental component ([PCr] = 38 s; VO_2_ = 39 s), as well as the relative amplitude of the slow component ([PCr] = 13.9%; VO_2_SC = 15.3%) of muscle [PCr] and VO_2_ during high-intensity exercise. It has been proposed that progressive intramuscular depletion [PCr] during exhaustive exercise performed within severe intensity domain provides the appropriate stimulus to oxidative phosphorylation, determining the development of VO_2_SC and, consequently, the attainment of VO_2_max (Rossiter et al., 2002). Thus, both creatine phosphate depletion and development of the VO_2_SC seem to be intimately associated with endurance performance during constant-work rate exercise (CWR) performed within the severe intensity domain.

While this scenario is well established during CWR exercise, very little information is available during intermittent exercise, which has been considered an important tool in training programs aiming to improve aerobic fitness in health and in disease (Laursen and Jenkins, 2002; Hwang et al., 2011). Indeed, intermittent exercise can improve performance comparing to CWR during high-intensity exercise (Millet et al., 2003; Chidnok et al., 2012), since the former allows resynthesis of intramuscular substrates ([PCr]) and/or clearance of fatigue-related metabolites (i.e., reconstitution of W') (Chidnok et al., 2013). However, several aspects seem to influence endurance performance during high-intensity intermittent exercises. For instance, endurance performance is progressively shorter when the work-recovery “duty-cycle” (e.g., 10:20 s, 30:60 s, 60:120 s, and 90:180 s) (Turner et al., 2006) and/or exercise intensity performed during active recovery is increased (i.e., light, moderate, heavy and severe) (Chidnok et al., 2012). These aspects influence PCr kinetics (Chidnok et al., 2013) and hypothetically, the changes of the rates at which VO_2_ increases during high-intensity intermittent exercises (i.e., VO_2_SC). Indeed, Chidnok et al. (2012) have demonstrated that enhanced endurance performance during severe-intensity intermittent exercise could be explained by the reconstitution of W' during recovery intervals performed at lower-intensity domains (i.e., light and moderate). At this condition, the reconstitution of W' was associated with a blunted increase in both VO_2_ and integrated EMG with time, supporting the hypothesis that VO_2_SC kinetics influences endurance performance during intermittent exercise. However, as discussed above, endurance performance during severe intermittent exercise is markedly modulated by both work-recovery duration and exercise intensity performed during active recovery. Thus, the possible relationship between VO_2_SC and endurance performance during intermittent exercise performed with different durations (e.g., short vs. long) and recovery type (i.e., passive vs. active) remains elusive, and further studies are warranted.

However, an important issue must be considered when the possible influence of VO_2_SC on endurance performance is investigated. Knowing that work-recovery duration influences endurance performance during severe intermittent exercise (Turner et al., 2006), it appears appropriate to compare exercise duration before (short condition) and after (long condition) the emergence of VO_2_SC. However, many studies have verified that both the emergence and the amplitude of VO_2_SC (and possibly the [PCr]) present a large intra-individual variation (Murgatroyd et al., 2011; Barbosa et al., 2014b). Thus, it would be interesting to analyze the responses of VO_2_ kinetics and endurance performance during severe intermittent exercise, with both the duration of exercise and recovery periods being determined based on the individual VO_2_SC kinetics response.

Thus, the current study was undertaken to compare the endurance performance and VO_2_SC kinetics during high-intensity intermittent exercise performed with different work:recovery duration (short vs. long) and recovery types (passive vs. active) in active individuals. It was hypothesized that: (a) endurance performance would be improved during the exercise with passive recovery, regardless of the duration of the repetition, and; (b) endurance performance would be improved during the intermittent exercise with short duration, regardless of the recovery type. We also hypothesized that the possible interaction between exercise duration and recovery type during intermittent high intensity exercise would influence the changes to the rates at which VO_2_ increases with time (VO_2_SC kinetics) and consequently, endurance performance.

## Materials and methods

### Subjects

Eighteen male students (24.7 ± 4.1 years; 80.5 ± 12.5 kg; 178.1 ± 7.6 cm) that were physically active but did not participate in any regular physical exercise or sport program volunteered for the study. All participants were healthy and free of cardiovascular, respiratory, and neuromuscular disease. All risks associated with the experimental procedures were explained prior to involvement in the study and each participant signed an informed consent form. The study was performed according to the Declaration of Helsinki and the protocol was approved by the University's Ethics Committee.

### Experimental design

The participants were instructed to report to the laboratory at the same time of the day (±2 h) on four separate occasions within a period of 2–3 week. Firstly, each volunteer performed an incremental test until exhaustion to determine the lactate threshold (LT), VO_2_max and the intensity associated with VO_2_max (IVO_2_max). Thereafter, the volunteers were divided into two groups: passive recovery (PR) and active recovery (AR) with similar IVO_2_max values. They performed the following protocols, on different days: (1) a total of two repetitions of square-wave transitions from rest to a power corresponding to 95% of the IVO_2_max to determine the parameters of VO_2_ kinetics. Each bout was separated by 60 min of passive rest. The VO_2_ responses to the two severe exercise bouts were averaged before the analysis to reduce the breath-to-breath noise and enhance confidence in the parameters derived from the modeling process (Lamarra et al., 1987) and; (2) two intermittent exercises, with the duration of the repetitions calculated from the onset of the exercise to the beginning of the VO_2_SC (Short) or to the half duration of the VO_2_SC (Long). The interval between the experimental sessions was 48–72 h. The participants were instructed to arrive at the laboratory in a rested and fully hydrated state at least 3 h post-prandial. They were also asked not to perform any strenuous activity during the day before each test.

### Procedures

#### Incremental test

Each participant performed an incremental exercise test to obtain volitional fatigue on an electronically braked cycle ergometer (Excalibur sport, Groningen, Netherlands) to determine the participant's LT, VO_2_max, and IVO_2_max. The incremental protocol started at a power output of 35 W, with increasing increments of 35 W every 3 min. Previous studies have demonstrated no differences in VO_2_max between incremental tests involving 1- or 3-min stage durations (Bentley and McNaughton, 2003; Roffey et al., 2007; Adami et al., 2013). The pedal cadence was kept constant (70 rpm) (Marsh and Martin, 1997). Throughout the tests, the respiratory and pulmonary gas-exchange variables were measured using a breath-by-breath gas analyzer (Quark PFTergo, Cosmed, Italy). The VO_2_max was defined as the highest average 15-s VO_2_ value recorded during the incremental test. IVO_2_max was defined as the power output at which the VO_2_max occurred. At the end of each stage, an earlobe capillary blood sample (25 μL) was collected into an eppendorf tube and analyzed for its lactate concentration ([La]) using an automated analyzer (YSI 2300 STAT, Yellow Spring, Ohio, USA). Plots of the blood [La] against the power output and VO_2_ were given to two independent reviewers, who determined LT as the first sudden and sustained increase in the blood lactate level above the resting concentrations.

#### Constant-workload exercise

The participants performed two exercise transitions at 95% IVO_2_max, separated by 60 min of rest. The first transition lasted 6 min and was conducted to determine the VO_2_ kinetics. The second transition was conducted until voluntary exhaustion to determine the VO_2_ kinetics (first 6 min) and the tlim (time to exhaustion). The protocol began with a 5 min warm-up at 50% IVO_2_max and was followed by a 7 min of passive rest. Then, the participants performed 3 min of unloaded cycling at 20 W, followed by a step change in the power output to 95% IVO_2_max. The pedal cadence was kept constant at 70 rpm. The second transition was terminated when the participant could not maintain a cadence of >65 rpm for >5 s despite verbal encouragement. The end-exercise VO_2_ was defined as the mean VO_2_ measured during the final 15 s of exercise. For the determination of [La] peak, capillary blood samples were collected 1, 3, and 5 min after the exercise, as previously described.

#### Intermittent exercises

**Figure 1:**
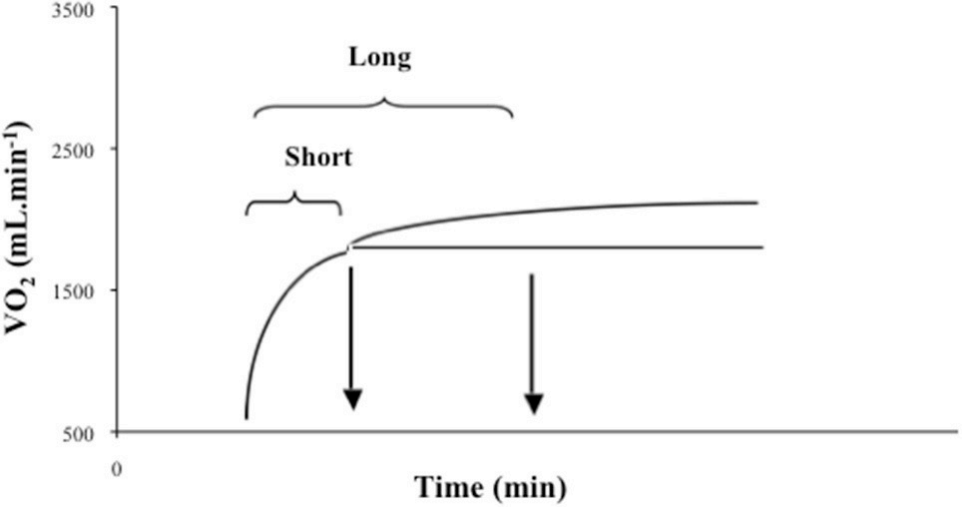
**Definition of the work intervals of the Short (beginning of the slow component) and Long (half duration of the slow component) intermittent protocols**.

The intermittent exercises were performed at 95% IVO_2_max, with the duration of the repetitions calculated from the onset of the exercise to the beginning of the VO_2_SC (i.e., time delay before the onset of the development of the VO_2_SC—Short) or the half duration of the VO_2_SC (i.e., 50% of the difference between the Short work interval duration and the time to achieve VO_2_max—Long) (Figure 1). The recovery was passive (PR) or active (AR) (50% IVO_2_max), with duration corresponding to the half of the repetition (effort:recovery ratio of 2:1). The exercises were performed until voluntary exhaustion. The criterion of exhaustion used was the same used for the constant-workload exercise. The end-exercise VO_2_ was defined as the mean VO_2_ measured during the final 15 s of exercise. If the duration of the last repetition was shorter than 90 s, the highest value of the previous bout was considered, to avoid underestimating the VO_2_ value.

#### Modeling of VO_2_ during constant-workload exercise

The breath-by-breath data from each exercise were manually filtered to remove outlying breaths, which were defined as breaths ±3 SD from the adjacent five breaths. The breath-by-breath data were interpolated to give second-by-second values. For CWR, the two transitions were then time aligned to the start of the exercise and averaged to enhance the underlying response characteristics. The first 20 s of data after the onset of exercise (i.e., the phase I response) (Whipp and Rossiter, 2005) were deleted, and the biexponential model was used to analyze the VO_2_ response to severe exercise, as described by the following equation:

(1) $$\begin{array}{l} {\text{VO}}_{2}(\text{t})={\text{VO}}_{2}\text{baseline}+\text{Ap }[1-{\text{e}}^{-(\text{t}-\text{TDp})/}\tau^{\text{p}}]\\ \;+\text{ As }[1-{\text{e}}^{-(\text{t}-\text{TDs})/}\tau^{\text{s}}] \end{array}$$

where: VO_2_(t) is the absolute VO_2_ at a given time t; VO_2_baseline is the mean VO_2_ in the baseline period; Ap, TDp, and τp are the amplitude, time delay, and time constant, respectively, describing the phase II increase in VO_2_ above baseline; and As, TDs, and τs are the amplitude of, time delay before the onset of, and time constant describing the development of the VO_2_SC, respectively. An iterative process was used to minimize the sum of the squared errors between the fitted function and the observed values. VO_2_baseline was defined as the mean VO_2_ measured over the final 60 s of exercise preceding the step transition to severe exercise. The amplitude of the VO_2_SC was determined as the increase in VO_2_ from TDs to the end of the modeled data (defined as As'). The end-exercise VO_2_ was defined as the mean VO_2_ measured over the final 15 s of exercise. The TD identified from Equation 1 was utilized to individualize the duration of the repetitions performed during short and long protocols (please see Section Intermittent exercises) and to estimate the VO_2_SC kinetics [i.e., the slow component trajectory (L.min^−^^2^)], as described below.

In addition, a single-exponential model without time delay, with a fitting window commencing at *t* = 0 s (equivalent to the mean response time), was used to characterize the kinetics of the overall VO_2_ response to exercise. The following equation describes this model:

(2) $${\text{VO}}_{2}(\text{t})={\text{VO}}_{2\text{baseline}}+\text{A }[1-{\text{e}}^{-(\text{t}/}\tau^{)}]$$

where: VO_2_(t) represents the absolute VO_2_at a given time t, VO_2_baseline represents the mean VO_2_ measured over the final 60 s of baseline pedaling, and A and τ represent the amplitude and time constant, respectively, which describe the overall increase in VO_2_ above the baseline. The VO_2_ was assumed to have essentially reached its maximal value when the value of [1–e^−(*t*/τ)^] from Equation 2 was 0.99 (i.e., when *t* = 4.6 × τ); it was assumed at this time that VO_2_ was at its maximal value. Therefore, for each exercise, the time to achieve VO_2_max (TAVO_2_max) was defined as 4.6 × τ. VO_2_SC kinetics [i.e., the slow component trajectory (L.min^−2^)] was also estimated by calculating the slope of the VO_2_ response using linear regression analysis (Chidnok et al., 2012). The data obtained before TDs (determined from Equation 1) were deleted to remove the influence of the fundamental response phase, and thereafter, VO_2_ values at 60-s intervals were determined until reaching the TAVO_2_max value and were fitted using the following equation:

(3) $${\text{VO}}_{2}=ax+b$$

where: x represents the time, a represents the slope, and b represents the y-intercept.

#### Modeling of VO_2_ during intermittent exercise

VO_2_SC kinetics [i.e., the slow component trajectory (L.min^−^^2^)] was estimated by calculating the slope of VO_2_ response using linear regression analysis (Chidnok et al., 2012). Final VO_2_ values (i.e., the average VO_2_ during 15 s) of each work cycle during intermittent exercise were determined up to the last completed cycle and fit using the Equation 3.

### Statistical analysis

The data are presented as means ± SD. The normality of data was checked by the Shapiro-Wilk test. A 2 × 3 two-way factorial analysis of variance (group vs. exercise condition), with repeated measures for the exercise condition factor (CWR vs. Short vs. Long) was used to analyze the VO_2_, tlim, slope VO_2_, [La] and HR data. When a significant interaction was found, follow-up analyses were performed using Tukey HSD test. The significance level was set at *p* < 0.05, and effect sizes were calculated using partial eta-squared (η^2^). All analyses were completed using the Statistical Package for the Social Sciences (SPSS v.20.0, SPSS Inc., Chicago, IL, USA).

## Results

Table 1 presents the mean ± SD values of the variables obtained during the incremental test for both PR and AR groups. No significant difference was found between the groups (*p* > 0.05).

**Table 1:** **Mean ± SD values of the variables obtained during the incremental test for both passive (PR) and active (AR) recovery groups**.

	**PR (*N* = 9)**	**AR (*N* = 9)**
VO_2_max (mL.min^−1^)	3220.4±271.8	3332.4±499.1
IVO_2_max (W)	250.3±25.5	266.9±44.1
P95% (W)	235.7±23.0	252.6±42.7
LT (W)	106.0±31.3	133.1±59.0
LT (%IVO_2_max)	41±11	48±16

*VO_2_max, maximal oxygen uptake; IVO_2_max, intensity at VO_2_max; P95%, power output relative to 95% IVO_2_max; LT, lactate threshold*.

**Figure 2:**
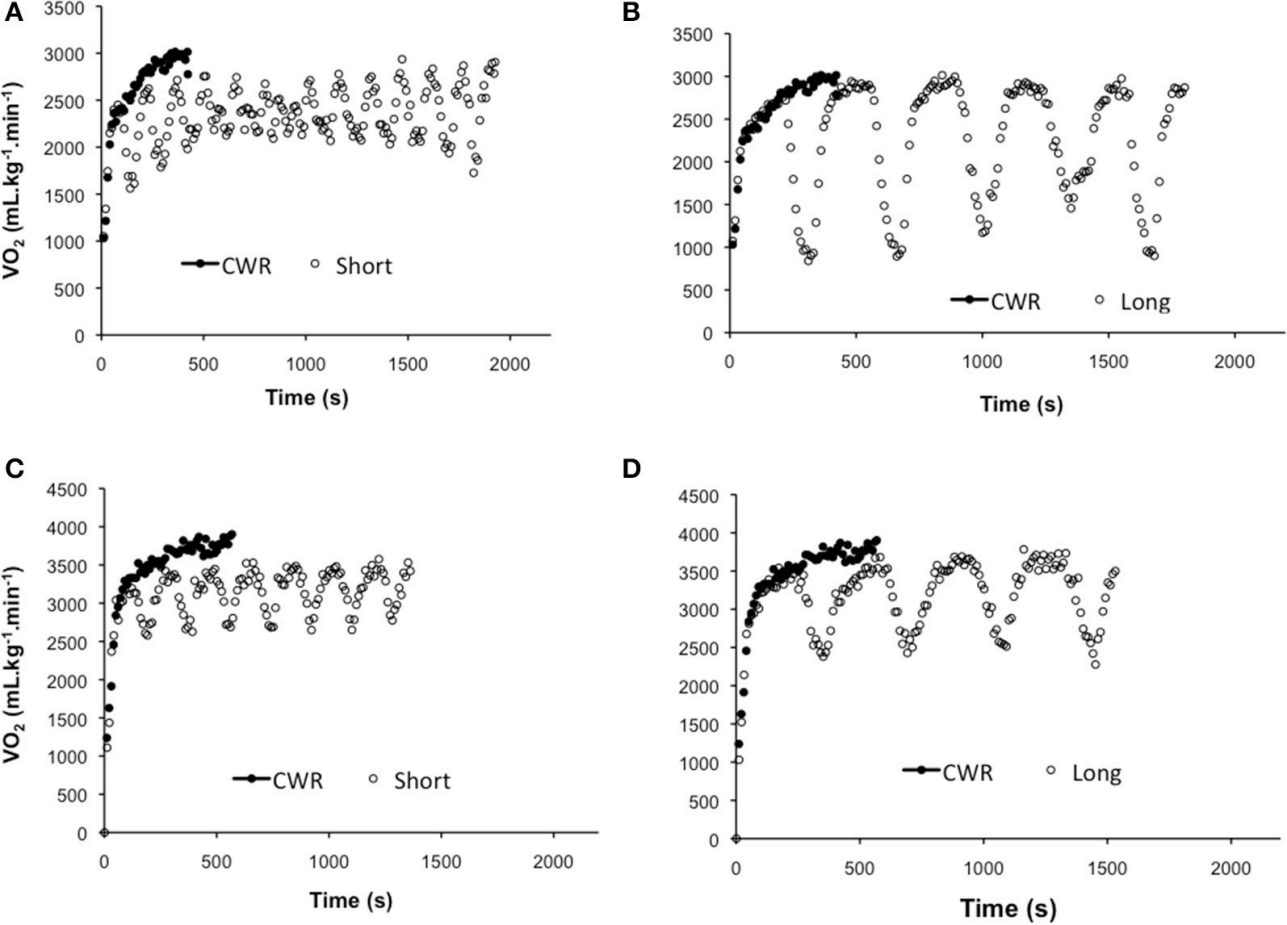
**Pulmonary oxygen uptake (VO_2_) response of a representative subject to constant-work rate (CWR) exercise (closed circles) compared with intermittent exercise (open circles) performed with passive** (**A**, short and **B**, long) and active (**C**, short and **D**, long) recovery.

The VO_2_ response profiles of a representative subject obtained during the different exercise conditions for both PR and AR groups are depicted in Figure 2. Based on the VO_2_ kinetics parameters obtained during CWR, the repetition duration for the Short (PR = 105 ± 29 s; AR = 132 ± 39 s) and Long (PR = 252 ± 50 s; AR = 253 ± 56 s) tests were not significantly different between the groups (*p* > 0.05).

**Figure 3:**
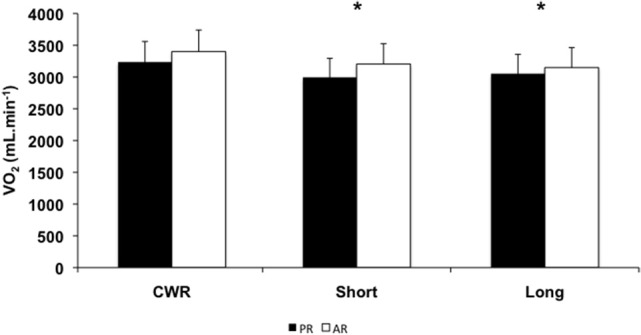
**Mean ± SD values of the end-exercise VO_2_ obtained during the exercise performed in different conditions for passive (PR) (*N* = 9) and active (AR) (*N* = 9) groups**. CWR—constant-work-rate exercise; ^*^p < 0.05 in relation to CWR.

Figure 3 presents the mean ± SD values of end-exercise VO_2_ measured during the different exercise conditions for both PR and AR groups. There was a significant main effect for the exercise condition on end-exercise VO_2_ values (*F* = 5.47, *p* = 0.009, η^2^ = 0.25), but no effect of group (*F* = 1.53, *p* = 0.23, η^2^ = 0.08) or interaction was detected (*F* = 1.25, *p* = 0.29, η^2^ = 0.07). The end-exercise VO_2_ values obtained during CWR (PR = 3236.9 ± 405.8 mL.min^−1^; AR = 3488.6 ± 415.9 mL.min^−1^) were higher than those attained during Short (PR = 2995.2 ± 337.7 mL.min^−1^; AR = 3205.7 ± 447.2 mL.min^−1^) and Long (PR = 3053.3 ± 276.1 mL.min^−1^; AR = 3149.6 ± 476.3 mL.min^−1^) tests (*p* < 0.05).

The mean ± SD values of tlim and VO_2_ slope during CWR and intermittent exercises for the PR and AR groups are presented in Table 2. A group vs. exercise condition interaction (*F* = 11.08, *p* = 0.000, η^2^ = 0.40) indicated longer tlim obtained during intermittent exercises (Short and Long) than CWR for both groups (*p* < 0.05). Considering the duration of the work and recovery type, tlim at Short was significantly longer than at Long only for the PR group (*p* < 0.05). Group effect (i.e., PR vs. AR) was significant only when comparing the Short intermittent protocols (*p* < 0.05), with no significant difference for Long conditions (*p* > 0.05). There was a significant main effect for the exercise condition on VO_2_ slope values (*F* = 95.98, *p* < 0.000, η^2^ = 0.90), but no group effect (*F* = 1.86, *p* = 0.19, η^2^ = 0.16) or interaction was detected (*F* = 0.02, *p* = 0.99, η^2^ = 0.01). VO_2_ slope was significantly greater at CWR than Short and Long conditions (*p* < 0.05).

**Table 2:** **Mean ± SD values of the time to exhaustion (tlim) and the slope of the oxygen uptake response (Slope) during the constant-work-rate (CWR) and intermittent exercise conditions (Short and Long), for passive (PR) and active (AR) recovery groups**.

	**PR (*N* = 9)**			**AR (*N* = 9)**			**Significance**
	**CWR**	**Short**	**Long**	**CWR**	**Short**	**Long**	
tlim (s)	540	1523	984	489	902	886	^*^F = 11.08
	116	411^‡^^,^ ^†^	260^‡^	84	239^‡^^,^^**^	254^‡^	*p* = 0.000
Slope (L.min^−2^)	0.16	0.01	0.02	0.16	0.03	0.01	^++^*F* = 5.34
	0.04	0.01^‡^	0.01^‡^	0.04	0.04^‡^	0.01^‡^	*p* = 0.01

* Group vs. condition interaction;

‡ p < 0.05 relative to the CWR condition;

† p < 0.05 relative to the Long condition;

** p < 0.05 relative to the Short condition;

++ *Main effect of exercise condition*.

The mean ± SD values of [La] and HR during CWR and intermittent exercises for the PR and AR groups are presented in Table 3. There was a significant main effect for the exercise condition on [La] values (*F* = 4.72, *p* = 0.01, η^2^ = 0.22), but no effect of group (*F* = 0.05, *p* = 0.81, η^2^ = 0.04) or interaction was detected (*F* = 1.76 *p* = 0.18, η^2^ = 0.09). The [La] was significantly lower at Short than CWR and Long condition (*p* < 0.05). A group *vs*. exercise condition interaction (*F* = 5.00, *p* = 0.01, η^2^ = 0.23) indicated that HR was lower during Short than Long and CWR only for the PR group (*p* < 0.05).

**Table 3:** **Mean ± SD values of the blood lactate concentration ([La]) and heart rate (HR) during the constant-work-rate (CWR) and intermittent exercise conditions (Short and Long), for passive (PR) and active (AR) recovery groups**.

	**PR**			**AR**			**Significance**
	**CWR**	**Short**	**Long**	**CWR**	**Short**	**Long**	
[La] (mM)	12.4	10.3	12.1	11.2	10.9	11.8	^++^*F* = 4.72
	2.83	3.70	3.02	2.35	2.65	2.78	*p* = 0.01
HR (bpm)	177 14	170 15^†^	177 11	184 7	183 6	186 5	^*^F = 5.00 *p* = 0.01

* Group vs. condition interaction;

† p < 0.05 relative to CWR and Long conditions;

++ *Main effect of exercise condition*.

## Discussion

This, we believe, is the first study to compare the endurance performance and VO_2_SC kinetics during severe-intensity intermittent exercise performed with different durations and recovery types in active individuals. The data demonstrate that endurance performance during severe-intensity intermittent exercise is negatively influenced by active recovery only during shorter (~120 s) intermittent exercise. Interestingly, slopes describing the increases in VO_2_ with time (i.e., VO_2_SC) and end-exercise VO_2_ were reduced during intermittent exercise (i.e., CWR vs. intermittent exercise). However, VO_2_ kinetics (VO_2_SC and end-exercise VO_2_) were similar between work:recovery duration (short vs. long) and recovery type (passive vs. active) analyzed in the present study, therefore rejecting our original hypothesis. Thus, the relationship between VO_2_ kinetics (VO_2_SC and end-exercise VO_2_) and endurance performance observed during CWR exercise (Jones et al., 2010; Barbosa et al., 2014a) seems to be differently regulated during severe-intensity intermittent exercise.

It has been widely reported that endurance performance during high-intensity intermittent exercise is improved when compared with CWR exercise (Demarie et al., 2000; Millet et al., 2003; Chidnok et al., 2012). However, both endurance performance and metabolic response are influenced by the characteristics of the protocol utilized during high-intensity intermittent exercise. Turner et al. (2006) analyzed the influence of duty cycle duration with the same work:recovery ratio (10:20 s, 30:60 s, 60:120 s, and 90:180 s) on pulmonary gas exchange and blood lactate dynamics during intermittent cycling exercise performed at 120% IVO_2_max. At this condition, a greater metabolic response (elevated blood lactate concentration and attainment of VO_2_max) and exercise intolerance (i.e., subjects could not complete 30 min of exercise) were observed only for the longer duty cycles (i.e., 60:120 s, and 90:180 s). Although our intermittent exercise protocol presents different characteristics (e.g., work:recovery = 2:1 and exercise intensity = 95% IVO_2_max), it was also verified a reduced endurance performance during longer duty cycles performed with passive recovery. The intramuscular PCr concentration ([PCr]) kinetics both during and following high-intensity exercise presents a curvilinear profile and seems to be closely linked with VO_2_ kinetics (Rossiter et al., 2002). For instance, under the conditions of the present study, is very likely that the amplitude of [PCr] restoration during the 240 s recovery intervals (Long protocol) was not doubled than what was presented when 120 s periods of recovery (Short protocol) were allowed. Moreover, Chidnok et al. (2013) demonstrated that [PCr] restoration become longer as the intermittent protocol continued. Thus, [PCr] is progressively lower immediately before each repetition, particularly when duty-cycle duration is lengthened. The metabolites generated by muscle contraction at this condition, such as Pi, ADP, and AMP, increase glycolytic flux and consequently, glycolytic H^+^ (Adams et al., 1990; Conley et al., 1997) and lactate (Karpatkin et al., 1964) production. Low values of muscle [PCr] and pH (i.e., high values of [H^+^]) and consistently high values of [Pi] and [ADP] have been associated with fatigue development during high-intensity exercise (Jones et al., 2008; Vanhatalo et al., 2010).

Another factor that can influence both endurance performance and metabolic response is the activity pattern performed during the recovery intervals between each bout (Chidnok et al., 2012). Using the CP model, Chidnok et al. (2012) demonstrated that endurance performance during intermittent exercise was enhanced only when the recovery intervals were performed below CP. Active recovery performed below CP allows a partial PCr reconstitution and/or clearance of fatigue-related metabolites (Chidnok et al., 2013), with the former being apparently more important to enhance endurance performance during high-intensity intermittent exercise. Indeed, both endurance performance (Chidnok et al., 2012) and PCr reconstitution (Chidnok et al., 2013) are higher during intermittent exercise with passive recovery than during active recovery performed bellow CP condition. Thus, a lower PCr reconstitution can explain, at least in part, the impaired endurance performance during short condition performed with active recovery, as observed in the present study.

However, a different scenario emerges from the data obtained during the Long intermittent exercise protocol. At this condition, endurance performance was not modified by the active recovery periods. Two different mechanisms, which can occur simultaneously, could help explain this phenomenon. Firstly, the negative influence of active recovery on PCr reconstitution could be time-dependent, i.e., longer duty-cycle could allow more similar PCr reconstitution than a shorter one. The curvilinear PCr recovery profile supports this hypothesis (Harris et al., 1976). Secondly, the clearance of lactate and H^+^ ions within muscles might be higher during the longer duty-cycle. A higher muscle pH can reduce, directly or indirectly (a more favorable metabolic milieu for PCr reconstitution), fatigue during high-intensity exercise. Alternatively, it is possible that [PCr] kinetics both during and following high-intensity intermittent exercise would contribute progressively less to endurance performance when the duty-cycle duration is lengthened.

The end-exercise VO_2_ was not significantly different between CWR exercise and VO_2_max measured during the incremental test. This is consistent with the fact that exhaustive exercise performed within the severe intensity domain (i.e., above CP) is characterized by the development of the VO_2_SC, which is truncated at VO_2_max. Some interventional (e.g., endurance training and priming exercise) (Jones et al., 2007; Caritá et al., 2014) and correlational studies (Barbosa et al., 2014a) have produced evidences that both VO_2_ kinetics (a proxy for intramuscular PCr kinetics) (Rossiter et al., 2002) and VO_2_max attainment is related to endurance performance during high-intensity exercise. Thus, it was hypothesized that VO_2_SC trajectory, which reflects the interaction between VO_2_SC and VO_2_max attainment, could explain the endurance performance during high-intensity intermittent exercise. Indeed, it was demonstrated that VO_2_SC trajectory was faster during CWR exercise than during intermittent exercise, regardless of duration and recovery type. However, similar to the results found by Chidnok et al. (2012), VO_2_SC trajectory was not significantly different among intermittent exercise, and end-exercise VO_2_ was lower during these conditions than at CWR exercise. Thus, substrate utilization/accumulation, VO_2_ kinetics (VO_2_SC trajectory and end-exercise VO_2_) and endurance performance during high-intensity exercise seem to present different relationship during CWR and intermittent exercise. Priming high-intensity exercise has previously been reported to reduce the amplitude of VO_2_SC and an increase in apparent W' during subsequent exercise (Caritá et al., 2014, 2015; Dekerle et al., 2015). In this context, each preceding intermittent exercise bout may have “primed” the muscle (i.e., reduces the amplitude of VO_2_SC and/or raise the W') during subsequent bouts. These modifications are consistent with enhanced endurance performance, and could help to explain the apparently different metabolic regulation imposed by the interaction between intervals duration and recovery type during intermittent exercise.

Our experimental protocol (i.e., exercise intensity, work:recovery durations and recovery types) was specifically designed to investigate the hypothetical association between intermittent endurance performance and VO_2_SC kinetics. Similar to previous studies (Caputo and Denadai, 2008; Barbosa et al., 2014a), both CWR and intermittent exercise were performed at 95% IVO_2_max. As demonstrated in the present study, exhaustive exercise performed at this intensity is characterized by the development of the VO_2_SC and VO_2_max attainment. Some studies have utilized the “percentage delta” (for details please see Lansley et al., 2011) aiming to select a predetermined exercise intensity domain (i.e., heavy or severe) and/or to standardize the exercise intensity between subjects. Indeed, when compared to a more traditional method (e.g., %VO_2_max), this approach allows a lower inter-subject variability of physiological responses to CWR exercise (Lansley et al., 2011). However, for the first time, the present study have normalized the wok:recovery durations based on the individual VO_2_SC kinetics response. Thus, we are confident that the inter-subject variability of physiological responses during the intermittent exercise was attenuated. Finally, this study presented a possible limitation, since the effect of passive and active recovery on intermittent exercise was analyzed using 2 different groups of active individuals. Hypothetically, this experimental design could be influenced by the individual variability on both endurance performance and VO_2_SC kinetics. However, PR and AR groups have presented similar data during incremental (VO_2_max, IVO_2_max, 95% IVO_2_max and LT) and CWR exercise (endurance performance and VO_2_SC kinetics). Therefore, the possibility of inter-subject variability influencing the recovery types comparisons was probably reduced. This limitation comes from the heavy testing required to be undertaken by each subject to test our research hypothesis. It is important to note that a short-term training program (6 sessions) involving high-intensity exercise (repeated all-out sprint training) have reduced the amplitude of the VO_2_SC and increased tolerance to high-intensity exercise in recreationally active subjects (Bailey et al., 2009). Thus, if a repeated measures design has been utilized in our experimental approach, a confounding factor could be added to our analysis, since the volunteers would have to perform 6 bouts of severe-intensity exercise.

## Conclusion

The present study showed that under our experimental conditions (i.e., exercise intensity, work:recovery durations and recovery type), intermittent exercise enhances endurance performance during severe-intensity exercise, independently of intervals duration and recovery type. Passive recovery is superior in relation to active recovery to enhance endurance performance only during shorter duty-cycles. Although VO_2_SC trajectory is attenuated during high-intensity intermittent exercise, its alteration does not seem to explain the interaction effects of intervals duration and recovery type on endurance performance. Moreover, the end-exercise VO_2_ was lower during intermittent exercise than at CWR exercise. Thus, severe-intensity intermittent exercise performed with different intervals duration and recovery type seems to modify the relationship between endurance performance and VO_2_ kinetics observed during CWR exercise. Further studies using a repeated measures design are required to examine the effect of severe-intensity intermittent exercise on both endurance performance and VO_2_SC in trained individuals. A threshold in the duration of the recovery, from which PCr resynthesis and/or W' reconstitution would be less affected by active recovery could be identified. This can help to explain and confirm our main results, giving support to elaborate a more sophisticate interval training programs for different populations.

## Author contributions

Study design: BD and CG. Data acquisition and analysis: LB, BD, and CG and Writing the paper: LB, BD, and CG.

## Funding

Supported by Fundação de Amparo à Pesquisa do Estado de São Paulo (FAPESP) (grant 2009/07700-2 and grant 2016/22907-6), Conselho Nacional de Desenvolvimento Científico e Tecnológico (CNPq) and Fundação para o Desenvolvimento da Unesp (FUNDUNESP).

### Conflict of interest statement

The authors declare that the research was conducted in the absence of any commercial or financial relationships that could be construed as a potential conflict of interest.
